# Adequação Alimentar de Indivíduos com Doença Cardiovascular Conforme Diretrizes Clínicas no Programa Alimentar Brasileiro Cardioprotetor

**DOI:** 10.36660/abc.20230705

**Published:** 2024-07-31

**Authors:** Luciana Brito, Viviane Sahade, Aline Marcadenti, Camila Ragne Torreglosa, Bernardete Weber, Ângela Cristine Bersch-Ferreira, Isa Galvão Rodrigues, Antônio Carlos Sobral Sousa, Adriana Barros Gomes, Josilene Maria Ferreira Pinheiro, Sandra Mary Lima Vasconcelos, Daniele Maria de Oliveira Carlos, José Albuquerque de Figueiredo, Clenise de Farias Dantas, Carla Daltro

**Affiliations:** 1 Programa de Pós-Graduação em Medicina e Saúde da UFBA Hospital Universitário Professor Edgard Santos Universidade Federal da Bahia Salvador BA Brasil Programa de Pós-Graduação em Medicina e Saúde da UFBA - Hospital Universitário Professor Edgard Santos - Universidade Federal da Bahia (UFBA), Salvador, BA – Brasil; 2 Empresa Brasileira de Serviços Hospitalares Salvador BA Brasil Empresa Brasileira de Serviços Hospitalares (EBSERH), Salvador, BA – Brasil; 3 Universidade Federal da Bahia Departamento de Nutrição Escola de Nutrição Salvador BA Brasil Universidade Federal da Bahia - Departamento de Nutrição da Escola de Nutrição da UFBA, Salvador, BA – Brasil; 4 Instituto de Pesquisa do Hcor São Paulo SP Brasil Instituto de Pesquisa do Hcor, São Paulo, SP – Brasil; 5 Real e Benemérita Associação Portuguesa de Beneficência São Paulo SP Brasil Real e Benemérita Associação Portuguesa de Beneficência, São Paulo, SP – Brasil; 6 Pronto Socorro Cardiológico Universitário de Pernambuco Recife PE Brasil Pronto Socorro Cardiológico Universitário de Pernambuco, Recife, PE – Brasil; 7 Universidade Federal de Sergipe Aracaju SE Brasil Universidade Federal de Sergipe, Aracaju, SE – Brasil; 8 Hospital Universitário Ana Bezerra Santa Cruz RN Brasil Hospital Universitário Ana Bezerra, Santa Cruz, RN – Brasil; 9 Universidade Federal de Alagoas Maceió AL Brasil Universidade Federal de Alagoas, Maceió, AL – Brasil; 10 Hospital de Messejana Fortaleza CE Brasil Hospital de Messejana, Fortaleza, CE – Brasil; 11 Universidade Federal do Maranhão São Luís MA Brasil Universidade Federal do Maranhão, São Luís, MA – Brasil; 12 Hospital Universiário Alcides Carneiro Universidade de Campina Grande Campina Grande PB Brasil Hospital Universiário Alcides Carneiro - Universidade de Campina Grande, Campina Grande, PB – Brasil; 13 Programa de Pós-Graduação em Medicina e Saúde da UFBA Departamento de Nutrição da Escola de Nutrição da UFBA Universidade Federal da Bahia Salvador BA Brasil Programa de Pós-Graduação em Medicina e Saúde da UFBA - Departamento de Nutrição da Escola de Nutrição da UFBA – Universidade Federal da Bahia, Salvador, BA – Brasil

**Keywords:** Doenças Cardiovasculares, Aterosclerose, Prevenção Secundária, Dieta, Ingestão de Alimentos

## Abstract

**Fundamento:**

Alcançar as metas nutricionais estabelecidas pelas sociedades científicas é um desafio constante e nem sempre alcançado.

**Objetivo:**

Investigar a adequação alimentar de indivíduos com doença cardiovascular (DCV), participantes do Programa Alimentar Brasileiro Cardioprotetor residentes da região Nordeste do Brasil, segundo as recomendações da Sociedade Brasileira de Cardiologia (SBC).

**Métodos:**

Análise transversal com dados do estudo de implementação da Dieta Cardioprotetora Brasileira (DICA BR) que avaliou indivíduos com DCV, atendidos em centros especializados em saúde cardiovascular em oito estados do Nordeste. O consumo alimentar foi obtido por recordatório alimentar de 24 horas e a adequação da dieta seguiu as recomendações da SBC. Foram considerados significantes valores de p < 0,05.

**Resultados:**

Foram estudados 647 pacientes, com média (desvio padrão) de idade de 63,1 (9,4) anos, sendo 50,2% do sexo feminino. Na avaliação da ingestão alimentar, observou-se baixa adequação de carboidratos (52,3%), proteínas (70,9%), lipídios (38,8%) e fibras (22,4%). Observou-se que a maioria das mulheres consumia dieta hipoproteica (59,2%) e idosos tinham maior inadequação no consumo de carboidratos (52,6%). Em relação a ingestão de sódio, os homens apresentaram maior ingestão (72,9%), enquanto os idosos apresentaram redução de 13%. Além disso, foi demonstrado que os homens ingeriam mais fibras (28,1%) e indivíduos com maior escolaridade tinham um consumo elevado de ácidos graxos saturados (70,5%).

**Conclusões:**

A maioria dos indivíduos não alcançou as metas dietoterápicas preconizadas para prevenção cardiovascular secundária. Os achados do presente estudo reforçam a necessidade de implementação de estratégias estruturadas, a fim de estimular hábitos alimentares saudáveis nesses indivíduos.

## Graphical abstract

**Figure:**
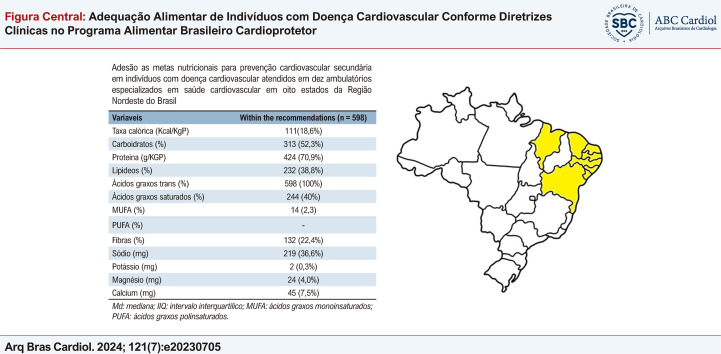

## Introdução

Dados da Organização Mundial de Saúde demonstram que as doenças cardiovasculares (DCV) lideram como a principal causa de mortalidade no mundo.^1^ No Brasil observa- se cenário semelhante, em que essas doenças foram responsáveis por aproximadamente 27% de todas as mortes, em 2019.^2^ Este dado é alarmante, pois aproximadamente 20% dos indivíduos que tiveram algum evento cardiovascular, sofrem o segundo evento um ano após o primeiro^3^ e a prevenção secundária é considerada ponto-chave para atingir de forma eficaz as metas terapêuticas de proteção cardiovascular.^4-7^

A prevenção cardiovascular secundária, é definida como o conjunto de estratégias preventivas, com objetivo de interromper a progressão da doença.^8^ Adoção de hábitos alimentares saudáveis e mudanças no estilo de vida, estão entre as estratégias importantes para a prevenção de eventos cardiovasculares recorrentes.^8,9^ Considerando esse contexto, foi desenvolvido o Programa Alimentar Brasileiro Cardioprotetor, denominado Dieta Cardioprotetora Brasileira (DICA BR),^10,11^ sendo um programa alimentar composto por prescrição dietética com adaptações da dieta mediterrânea através da valorização de alimentos factíveis para população brasileira, baseado nas recomendações nutricionais das diretrizes da Sociedade Brasileira de Cardiologia.^12^

Apesar dos estudos demonstrarem a importância da alimentação na prevenção cardiovascular secundária, alcançar as metas terapêuticas estabelecidas pelas sociedades científicas é um desafio constante e nem sempre atingido.^5^ Apesar do amplo conhecimento sobre a importância da redução de fatores de risco cardiovascular, uma elevada parcela de indivíduos com DCV não atinge adequadamente o controle destes fatores.^5,10^

Embora tenha sido observada mundialmente uma redução da mortalidade por DCV,^1^ as taxas de morte por essas doenças continuam elevadas em algumas regiões brasileiras, com destaque para a região Nordeste do país.^2^ Entretanto, os estudos que avaliam as medidas de prevenção secundária nesta região são escassos. Assim, o objetivo do presente estudo foi investigar a adequação alimentar de indivíduos com doença cardiovascular (DCV), participantes do Programa Alimentar Brasileiro Cardioprotetor residentes da região Nordeste do Brasil, segundo as recomendações da Sociedade Brasileira de Cardiologia.

## Métodos

### Desenho do estudo

Trata-se de um estudo transversal, parte de um trabalho mais amplo, conduzido com dados de pacientes do estudo de implementação da Dieta Cardioprotetora Brasileira (DICA BR).^11^ O processo de coleta de dados foi realizado no baseline entre 2013 a 2015, por nutricionistas previamente treinados. A descrição detalhada do desenho do estudo foi publicada anteriormente.^12^

### Amostra

A amostra foi composta por pacientes atendidos em dez centros de referência em saúde cardiovascular localizados na Região Nordeste do Brasil. Os critérios de inclusão adotados no estudo DICA BR foram idade igual ou superior a 45 anos, ambos os sexos e ter evidência de aterosclerose (doença arterial coronariana, doença cerebrovascular ou doença arterial periférica), atual ou nos últimos dez anos diagnosticada pelo médico assistente.

Foram considerados portadores de doença arterial coronariana (DAC) aqueles pacientes que apresentassem uma ou mais das seguintes características: DAC assintomática (história de angiografia coronariana ou angiotomografia coronariana com estenose aterosclerótica ≥70% do diâmetro de qualquer artéria coronária); DAC sintomática (história de angina: diagnóstico clínico, mesmo sem exames complementares; história de positividade ao teste de esforço); DAC tratada (realização prévia de angioplastia/Stent/revascularização) e infarto (história de infarto do miocárdio ou síndrome coronariana aguda; história de anormalidade no movimento segmentar da parede cardíaca na ecocardiografia ou um defeito segmentar fixo em cintilografia).

Por sua vez, o diagnóstico de doença cerebrovascular (Acidente Vascular Cerebral-AVC isquêmico/ Acidente Isquêmico Transitório-AIT/Acidente Vascular Encefálico-AVE), foi considerado quando o paciente apresentou um ou mais dos seguintes critérios: diagnóstico médico de AVC ou AIT; evidência de AVC prévio na tomografia computadorizada ou na ressonância nuclear magnética.

Em relação ao diagnóstico de doença arterial periférica (DAP), este foi considerando quando o indivíduo apresentou, um ou mais dos critérios a seguir: DAP assintomática (relação tornozelo/braço <0,9 de pressão arterial sistólica em qualquer perna em repouso; estudo angiográfico ou doppler demonstrando estenose >70% em uma artéria não cardíaca); DAP sintomática (claudicação intermitente); DAP tratada (cirurgia vascular para doença aterosclerótica); Amputação por causa arterial e aneurisma de aorta.

Os critérios de não inclusão foram: condição psiquiátrica e/ou neurocognitiva que impedisse a obtenção de dados; expectativa de vida inferior a seis meses; gravidez ou lactação; insuficiência hepática com história de encefalopatia ou anasarca; insuficiência renal com indicação de diálise; insuficiência cardíaca congestiva; transplante de órgãos; gastroplastia; cadeirante e participantes que apresentassem dificuldade de alimentação via oral.

### Coleta de dados

Os dados obtidos para este trabalho foram: tabagismo, sedentarismo, hipertensão arterial (HAS), diabetes mellitus (DM), dislipidemia, doença arterial coronariana tratada (DAC e DAP tratada) e aferição da pressão arterial, conforme descrito em literatura.^13^ Em relação das variáveis socioeconômicas, o nível de escolaridade foi classificado em menor (analfabeto ou ensino fundamental) ou maior nível (ensino médio ou superior). Quanto à classe social, esta foi classificada em menor (C, D e E) ou maior (A e B).^14^

### Exames laboratoriais

A análise dos exames laboratoriais foi realizada com o paciente em jejum de 12 horas e o não consumo de álcool nas 72 horas anteriores. Foram utilizados marcadores de risco cardiovascular tradicionais: triglicérides, colesterol total, lipoproteínas de alta densidade (HDL- c), lipoproteínas de baixa densidade (LDL-c) e colesterol não HDL, sendo classificados conforme recomendações preconizadas.^7,15^

### Avaliação antropométrica

Os dados de peso e altura foram utilizados para calcular a variável índice de massa corpórea (IMC). Para fins de análise, os indivíduos foram agrupados com presença de baixo peso, quando o IMC fosse inferior a 18,5kg/m^2^ e 23,0kg/m^2^; eutrófico com valores compreendidos entre 18,5-24,9kg/m^2^ e 23,0-27,9kg/m^2^; excesso de peso quando o IMC foi igual ou superior a 25,0kg/m^2^ e 28,0kg/m^2^, respectivamente em adultos e idosos. A obesidade foi definida com IMC igual ou superior a 30,0kg/m^2^, para ambos.^16,17^ A presença de obesidade central foi determinada a partir da medida da circunferência da cintura, conforme técnica e pontos de corte preconizados.^18^

### Avaliação da ingestão alimentar

Em relação à análise da ingestão alimentar foi utilizado o recordatório alimentar de 24 horas (R24H), sendo obtido segundo a metodologia de múltiplas passagens,^19^ com a utilização de um álbum fotográfico de medidas e porções de alimentos, objetivando aumentar a confiabilidade das informações coletadas. Para a análise da composição nutricional dos R24H obtidos, foi utilizado o programa Nutriquanti®, o qual prioriza as tabelas de composição nutricional brasileiras.

A quantidade do valor energético total (VET) e proteínas ingeridas foi expressa por quilograma de peso. Para indivíduos com excesso de peso e obesos foi considerado adequado o consumo energético na faixa de 20,0 a 25,0kcal/kgPatual/dia.^20^ Entretanto, diante da ausência de recomendação da Sociedade Brasileira de Cardiologia para ingestão de energia em indivíduos com aterosclerose com baixo peso e eutróficos, foi estabelecido como adequada 30,0 a 35,0kcal/KgP e 25,0 a 30,0kcal/KgP, respectivamente.^21^

A ingestão de macronutrientes (carboidratos e lipídeos) foi avaliada em percentual utilizando-se como referência o VET consumido. O consumo de proteína (igual ou superior a 0,8g/KgP),^20^ sódio (inferior a 2.000mg),^13^ carboidratos (45 a 60%), fibra alimentar (igual ou superior a 25g), lipídios (25 a 35%), ácidos graxos poliinsaturados (PUFA) de 5 a 10%, monoinsaturados (MUFA) 15%, saturados (AGsat) inferior a 7% e ausência de ácidos graxos trans.^7^

Quanto as adequações nutricionais referentes ao consumo de cálcio, magnésio e potássio, em virtude da ausência de recomendação especificas para indivíduos com DCV, estas foram consideradas de acordo com o sexo e a faixa etária com base nas recomendações de consumo de micronutrientes da EAR (*Estimated Average Requirements*), através da *Dietary reference intakes* (DRI).^22^

### Comitê de ética

O estudo foi conduzido de acordo com os princípios éticos e o protocolo do estudo foi aprovado pelo Comitê de Ética em Pesquisa com Seres Humanos do [removido para revisão cega por pares], bem como dos comitês de ética de cada centro colaborador. Todos os participantes assinaram o Termo de Consentimento Livre e Esclarecido (TCLE).

### Análise estatística

Para análise dos dados foi utilizado o programa Statistical Package for the Social Sciences (SPSS), versão 17. Os dados foram apresentados por frequência simples e relativa, média e desvio padrão (DP) ou mediana (Md) e intervalo interquartílico (IIQ), conforme natureza das variáveis. A normalidade das variáveis quantitativas foi testada utilizando a estatística descritiva, análise gráfica e testes de normalidade (Kolmogorov-Smirnov). As associações foram testadas por meio da razão de prevalência com respectivos intervalos de confiança de 95% (IC 95%), em que na análise de razão de prevalência foi atribuído o valor 1 a categoria de referência. Os indivíduos foram divididos em dois grupos, “dentro da recomendação sim ou não” para fins d e comparação, sendo utilizado o teste qui-quadrado de Pearson para comparação das proporções. Foram considerados significantes valores de p < 0,05.

## Resultados

Foram selecionados 654 indivíduos, sendo que sete não foram incluídos na amostra, por não terem comparecido a consulta inicial, totalizando 647 pacientes. Dos nove estados que compõem a região Nordeste do Brasil, apenas o estado do Piauí não participou do estudo. Os pacientes tinham média (DP) de idade de 63,1 (9,3) anos, com predominância do sexo feminino, de idosos, de indivíduos com baixo nível de escolaridade e sedentários (Tabela 1).

**Tabela 1:** – Características sociodemográficas, de estilo de vida, clínicas e antropométricas de 647 indivíduos com doença cardiovascular, acompanhado em ambulatórios de referência em atenção cardiovascular em oito estados do Nordeste do Brasil

Variáveis	Resultados
**Sociodemográficas e de estilo de vida**	
Idade (anos)*	63,1 (9,3)
Sexo feminino	325 (50,2%)
**Nível de escolaridade**	
Analfabeto	210 (37,6%)
Fundamental	211 (37,8%)
Médio	100 (17,9%)
Superior	37 (6,6%)
**Classe social**	
Classe A e B	112 (20,0%)
Classe C, D e E	447 (80,0%)
Sedentarismo	463 (73,3%)
Ex-tabagismo	342 (53,0%)
Tabagismo	27 (4,2%)
**Clínicas**	
Dislipidemia	625 (96,6%)
Doença arterial coronariana	599 (92,6%)
Hipertensão arterial	580 (89,6%)
Infarto agudo do miocárdio	294 (45,4%)
Diabetes mellitus	261 (40,3%)
Acidente vascular cerebral	111 (17,2%)
Doença arterial periférica	61 (9,4%)
Doença arterial coronariana tratada	425 (65,7%)
**Antropometria**	
Baixo peso	47 (7,3%)
Eutrofia	229 (35,7)
Excesso de peso	177 (27,6%)
Obesidade	188 (29,3%)
Obesidade abdominal	486 (77,0%)
**Alcance das metas terapêuticas**	
LDL-c	45 (7,8%)
Não-HDL-c	57 (9,8%)
Triglicérides	341 (58,6%)

*Dados em média e desvio padrão.

Na avaliação da ingestão alimentar, 49 indivíduos não tinham o registro alimentar na consulta basal, portanto, a análise da composição nutricional foi realizada em 598 indivíduos. Observaram-se inadequações do consumo de macronutrientes, fibras e micronutrientes, conforme descrito na Tabela 2.

**Tabela 2:** – Adesão as metas nutricionais para prevenção cardiovascular secundária em 598 indivíduos com doença cardiovascular atendidos em dez ambulatórios especializados em saúde cardiovascular em oito estados da Região Nordeste do Brasil

Variáveis	Resultados	Dentro das Recomendações
Taxa calórica (Kcal/KgP)*	24,7 (22,6 - 28,0)	111 (18,6%)
Carboidratos (%)	55,6 (10,6)	313 (52,3%)
Proteína (g/KgP)	1,0 (0,7 - 1,3)	424 (70,9%)
Lipídeos (%)	24,6 (7,6)	232 (38,8%)
Ácidos graxos trans (%)*	0,02 (0,01 - 0,03)	598 (100,0%)
Ácidos graxos saturados (%)	7,8 (5,7 - 10,6)	244 (40,8%)
MUFA (%)	6,9 (5,3 - 8,7)	14 (2,3%)
PUFA (%)	0,6 (0,5 - 0,8)	-
Fibras (g)	16,5 (11,4 - 23,2)	134 (22,4%)
Sódio (mg)	2.384,3 (1.720,6 - 3.180,6)	219 (36,6%)
Potássio (mg)	2.037,0 (1.491,1 - 2.649,3)	2 (0,3%)
Magnésio (mg)	182,2 (132,1 - 239,5)	24 (4,0%)
Cálcio (mg)	471,0 (271,7 - 757,1)	45 (7,5%)

*Md: mediana; IIQ: intervalo interquartílico; MUFA: ácidos graxos monoinsaturados; PUFA: ácidos graxos poliinsaturados.

As tabelas 3 e 4 descrevem a prevalência de inadequações no consumo alimentar e razões de prevalência, de acordo com gênero, faixa etária e variáveis sociodemográficas. O resumo dos achados do estudo pode ser observado na Figura Central.

**Tabela 3:** – Prevalência e razões de prevalência brutas de inadequações no consumo de calorias e macronutrientes, de acordo com gênero, faixa etária e variáveis sociodemográficas, de indivíduos com aterosclerose acompanhados em dez ambulatórios de referência em saúde cardiovascular em oito cidades da Região Nordeste

Variáveis	Taxa calórica inadequada				Consumo inadequado de Carboidratos				Baixo consumo proteico			
	n	p (%)	RP	IC 95%	n	p (%)	RP	IC 95%	n	p (%)	RP	IC 95%
**Sexo**												
Masculino	230	78,8	0,94	0,87 - 1,01	140	47,9	1,01	0,86 - 1,20	71	40,8	0,72	0,56 - 0,93
Feminino	256	83,9	1,00		145	47,4	1,00		103	59,2	1,00	
**Idoso**												
Sim	292	81,1	0,99	0,92 - 1,07	190	52,6	1,31	1,09 - 1,58	100	57,5	0,89	0,69 - 1,14
Não	194	81,9	1,00		95	40,1	1,00		74	42,5	1,00	
**Escolaridade**												
Menor	329	82,9	1,07	0,97 - 1,19	191	48,0	0,93	0,75 - 1,16	123	77,4	0,88	0,64 - 1,21
Maior	102	77,3	1,00		59	44,7	1,00		36	22,6	1,00	
**Classe social**												
Menor	350	82,9	1,08	0,97 - 1,21	203	48,1	0,91	0,71 - 1,15	129	81,1	0,91	0,65 - 1,27
Maior	82	76,6	1,00		47	43,5	1,00		30	18,9	1,00	

*AGSat: ácidos graxos saturados; RP: razão de prevalência.*

**Tabela 4:** – Prevalência e razões de prevalência brutas de inadequações no consumo de fibras, sódio e ácidos graxos saturados, de acordo com gênero, faixa etária e variáveis sociodemográficas, de indivíduos com aterosclerose acompanhados em dez ambulatórios de referência em saúde cardiovascular em oito cidades da Região Nordeste

Variáveis	Baixo consumo de fibras				Consumo elevado de sódio				Consumo elevado de AGSat			
	n	p (%)	RP	IC 95%	n	p (%)	RP	IC 95%	n	p (%)	RP	IC 95%
**Sexo**												
Masculino	210	71,9	0,87	0,79 - 0,95	213	72,9	1,34	1,19 - 1,52	176	60,3	1,04	0,91 - 1,18
Feminino	254	83,0	1,0		166	54,2	1,0		178	58,2	1,00	
**Idoso**												
Sim	284	78,7	1,04	0,95 - 1,13	216	59,8	0,87	0,77 - 0,98	207	57,3	0,92	0,81 - 1,06
Não	180	75,9	1,0		163	68,8	1,0		147	62,0	1,00	
**Escolaridade**												
Menor	313	78,6	1,09	0,97 - 1,23	245	61,6	0,87	0,76 - 1,00	225	56,5	0,82	0,71 - 0,95
Maior	95	72,0	1,0		93	70,5	1,0		91	68,9	1,00	
**Classe social**												
Menor	331	78,4	1,09	0,96 - 1,23	266	63,0	0,94	0,81 - 1,10	244	57,8	0,86	0,73 - 1,00
Maior	78	72,2	1,0		72	66,7	1,0		73	67,6	1,00	

*AGSat: ácidos graxos saturados; RP: razão de prevalência.*

## Discussão

No presente estudo foi verificado que a maior parte dos indivíduos apresentou elevada inadequação às metas dietoterápicas estabelecidas para prevenção cardiovascular secundária. Em outros estudos também foram demonstrados relevantes inadequações as metas nutricionais, corroborando os resultados apresentados.^23,24^ Além disso, foi verificado que indivíduos idosos tinham maior inadequação de consumo de carboidratos, aqueles com maior escolaridade tinham consumo elevado de AGsat, além do mais os homens apresentaram maior ingestão de sódio e as mulheres maior inadequação de fibras. Esses dados chamam atenção, pois indivíduos com essas características provavelmente apresentem baixa adequação alimentar. Vale ressaltar que as inadequações alimentares podem ter influenciado o não atendimento às metas terapêuticas relacionadas ao perfil lipídico,^7^ controle pressórico^13^ e glicêmico.^15^

A Sociedade Brasileira de Cardiologia e a *American Heart Association* recomendam que, é potencialmente benéfico a aplicação conjunta de estratégias relacionadas a terapia medicamentosa e não farmacológica no manejo da aterosclerose.^4,6,7^ Estudos anteriores investigaram a associação de padrões alimentares com eventos cardiovasculares reincidentes^11,25^e que a adoção dos hábitos alimentares saudáveis parece atuar sinergicamente com o controle dos fatores de risco cardiovasculares.^26^

O consumo alimentar “aterogênico”, evidenciado pelo consumo elevado de AGsat e sódio, predominou nos indivíduos estudados. Evidências demonstram possíveis nutrientes apontados como fatores de risco para DCV, dentre os quais podem ser citados: elevado consumo de sódio, AGsat, gordura trans^27^ e carboidratos refinados.^28^ Em contraposição, a redução do consumo de alimentos fontes desses nutrientes, tem demonstrado ser de extrema importância na prevenção e controle das DCV.^29^

Ao analisar a ingestão alimentar da amostra estudada, observa-se que poucos indivíduos estavam consumindo a quantidade de calorias necessárias para o seu estado nutricional. A literatura demonstra possíveis fatores que dificultam atingir as recomendações nutricionais, dentre os quais: questões socioeconômicas^30^ e ausência de conhecimento ou má adesão as orientações nutricionais.^31^

É importante ressaltar que avaliar a qualidade da dieta é extremamente relevante. No presente estudo, apesar da maioria dos indivíduos apresentarem dieta com característica hipolipidica, observa-se elevado consumo de AGsat e baixo de ácidos graxos insaturados, corroborando outros autores.^32^ Estudos têm demonstrado que modificações qualitativas na composição lipídica da dieta parecem ter efeitos benéficos na redução de evento e mortalidade cardiovascular, apesar dos resultados serem conflitantes.^27^ Em uma metanálise, foram observados resultados conflitantes de dietas com redução de lipídeos e da substituição de AGsat por PUFA.^33^ Isso pode ser atribuído ao fato de que a troca de AGsat por ácidos graxos insaturados, além de diminuir o LDL-c, pode reduzir o HDL-c, ocorrendo de forma mais intensa ao substituir AGsat por MUFA.^34^

Entre estratos de maior escolaridade, o nível de instrução demonstrou estar associado à maior ingestão de AGsat. Sendo assim, ao analisarmos a escolaridade como medida indireta de renda, observa-se que indivíduos com nível socioeconômico mais favorável apresentam maior prevalência de inadequação de consumo de AGsat, corroborando outros autores.^35^ Neste contexto, cabe também ressaltar que, no presente estudo aproximadamente 30% dos indivíduos tinham dieta hipoprotéica. Sabe-se que esses alimentos habitualmente apresentam custo mais elevado e estão menos disponíveis entre os indivíduos de menor renda.^36^

Foi evidenciado que uma pequena parcela de indivíduos atingiu a recomendação de fibra alimentar, achado similar foi encontrado por outros estudos,^23,37^ possivelmente devido a adoção de hábitos alimentares pouco saudáveis, baixa qualidade do carboidrato ingerido^28^ e alto consumo de ultraprocessados identificado pelo consumo elevado de sódio.^37^ No que se refere às diferenças de consumo de fibras alimentares entre os sexos, as mulheres apresentaram maior prevalência de inadequação. Estudos têm demonstrado que o consumo de fibra alimentar reduz proporcionalmente na presença de piores condições socioeconômicas, o que possivelmente poderia justificar o baixo consumo evidenciado entre as mulheres.^36^ Uma metanálise demonstrou que a ingestão de fibras foi associada com uma redução benéfica no colesterol total, LDL-c e pressão arterial diastólica.^38^

O consumo elevado de sódio foi maior entre homens, achado semelhante ao de outros autores.^39^ Segundo a Pesquisa de Orçamento Familiar (POF), o consumo diário de sódio no Brasil é de aproximadamente 4.700 mg.^40^ Em virtude de a ingestão de sódio ser considerada um importante marcador de qualidade da dieta na prevenção cardiovascular secundária, reforça-se a necessidade de aplicação de ações de prevenção, visando redução de consumo de sódio.^24^

Ainda em relação ao consumo alimentar, o potássio, magnésio e cálcio são nutrientes essenciais, que desempenham importante papel na preservação do tônus vascular e contratilidade cardíaca.^23^ O consumo desses micronutrientes no presente estudo foi extremamente baixo, o que reflete a baixa qualidade da dieta, podendo ser justificada pelo consumo inadequado de alimentos fontes de vitaminas e minerais, além da predominância de indivíduos de baixo poder aquisitivo, favorecendo no menor acesso a uma alimentação mais variada e saudável.^36^

Entre as limitações deste estudo, pode-se citar a ausência de métodos mais precisos para avaliar o estado nutricional. Entretanto, o IMC e a circunferência da cintura foram avaliados conjuntamente, a fim de minimizar essa limitação. Ressalta-se que, esses indicadores apresentam baixo custo e fácil aplicação na prática clínica. Além disso, deve-se considerar que é possível que tenha ocorrido viés de memória, visto que o R24H é um instrumento dependente da memória do entrevistado. As limitações do uso deste instrumento residem no fato de que a ingestão alimentar em um único dia pode não refletir a dieta habitual do indivíduo. Entretanto, acreditamos que esses indivíduos apresentam baixa variabilidade de consumo alimentar, devido ao fato de a amostra ser composta predominantemente por indivíduos de classes sociais mais baixas.

É importante ressaltar que diante da ausência de recomendações especificas para indivíduos com DCV, a adequação da ingestão de micronutrientes foi realizada com base na DRI. Entretanto, essas recomendações foram desenvolvidas para uma população saudável e não brasileira, portanto os achados devem ser analisados com cautela.

A DICA BR é o primeiro estudo nacional trabalhando a regionalidade e alimentação relacionando-as com a saúde cardiovascular. Destaca-se que o programa é inédito no país, pois considera a cultura alimentar das regiões brasileiras. A adesão à hábitos alimentares saudáveis é um processo desafiador e dinâmico tanto para os pacientes quanto para os profissionais de saúde.^31^ Diante do êxito da Primeira Dieta Cardioprotetora Brasileira foi elaborado pelo Ministério da Saúde um manual de orientações para profissionais de saúde da Atenção Básica^41^ com orientações nutricionais para indivíduos portadores de fatores de risco cardiovasculares.

De fato, a intervenção dietética adequada permite uma melhor combinação de múltiplos alimentos e nutrientes, com propriedade cardioprotetora na prevenção secundária das DCV. Dessa forma, é importante estimular o maior consumo desses nutrientes, favorecendo o melhor controle dos fatores de risco cardiovascular. Destaca-se o fato desse trabalho ser um estudo regional, com objetivo de fornecer informações relevantes para profissionais de saúde sobre o perfil de indivíduos nordestinos com DCV, bem como identificar fatores que possam exercer influência na adequação nutricional na prevenção cardiovascular secundária.

## Conclusões

Os resultados do estudo indicaram que a maioria dos indivíduos não alcançou as metas dietoterápicas preconizadas para prevenção cardiovascular secundária, conforme a Sociedade Brasileira de Cardiologia. Dessa forma, tinham uma dieta pobre em fibras, em micronutrientes e de baixa qualidade nutricional, com elevada ingestão de gordura saturada e baixa de ácidos graxos insaturados, tudo isso predispondo a ocorrência de outros desfechos cardiovasculares. Foi identificado consumo excessivo de sódio, principalmente no sexo masculino, sendo um fator preocupante, pois esses indivíduos, devido ao risco cardiovascular muito elevado, requerem valores menores de consumo deste nutriente.
