# Supplementary material for: Adequação Alimentar de Indivíduos com Doença Cardiovascular Conforme Diretrizes Clínicas no Programa Alimentar Brasileiro Cardioprotetor
Source: Arq Bras Cardiol. 2024 Jul 31;121(7):e20230705. [Article in Portuguese] doi: 10.36660/abc.20230705 (PMC11341214; doi:10.36660/abc.20230705)
Supplement: Translation

# Translation: Dietary Adequacy of Individuals with Cardiovascular Disease According to Clinical Guidelines in the Brazilian Cardioprotective Nutritional (BALANCE)

**DOI:** 10.36660/abc.20230705i

**Authors:** Luciana Brito, Viviane Sahade, Aline Marcadenti, Camila Ragne Torreglosa, Bernardete Weber, Ângela Cristine Bersch-Ferreira, Isa Galvão Rodrigues, Antônio Carlos Sobral Sousa, Adriana Barros Gomes, Josilene Maria Ferreira Pinheiro, Sandra Mary Lima Vasconcelos, Daniele Maria de Oliveira Carlos, José Albuquerque de Figueiredo, Clenise de Farias Dantas, Carla Daltro

**Affiliations:** 1 Brazil Programa de Pós-Graduação em Medicina e Saúde da UFBA - Hospital Universitário Professor Edgard Santos - Universidade Federal da Bahia (UFBA), Salvador, BA – Brazil; 2 Brazil Empresa Brasileira de Serviços Hospitalares (EBSERH), Salvador, BA – Brazil; 3 Brazil Universidade Federal da Bahia - Departamento de Nutrição da Escola de Nutrição da UFBA, Salvador, BA – Brazil; 4 Brazil Instituto de Pesquisa do Hcor, São Paulo, SP – Brazil; 5 Brazil Real e Benemérita Associação Portuguesa de Beneficência, São Paulo, SP – Brazil; 6 Brazil Pronto Socorro Cardiológico Universitário de Pernambuco, Recife, PE – Brazil; 7 Brazil Universidade Federal de Sergipe, Aracaju, SE – Brazil; 8 Brazil Hospital Universitário Ana Bezerra, Santa Cruz, RN – Brazil; 9 Brazil Universidade Federal de Alagoas, Maceió, AL – Brazil; 10 Brazil Hospital de Messejana, Fortaleza, CE – Brazil; 11 Brazil Universidade Federal do Maranhão, São Luís, MA – Brazil; 12 Brazil Hospital Universiário Alcides Carneiro - Universidade de Campina Grande, Campina Grande, PB – Brazil; 13 Brazil Programa de Pós-Graduação em Medicina e Saúde da UFBA - Departamento de Nutrição da Escola de Nutrição da UFBA – Universidade Federal da Bahia, Salvador, BA – Brazil

**Keywords:** Cardiovascular Diseases, Atherosclerosis, Secondary Prevention, Diet, Eating

## Abstract

**Background:**

Achieving nutritional goals established by scientific societies is a constant challenge and not always achieved.

**Objective:**

To investigate the dietary adequacy of individuals with cardiovascular disease (CVD), participants in the Cardioprotective Brazilian Food Program residing in the Northeast region of Brazil, according to the recommendations of the Brazilian Society of Cardiology (SBC).

**Methods:**

Cross-sectional analysis with data from the study implementing the Brazilian Cardioprotective Diet (DICA BR), which evaluated individuals with CVD treated in specialized cardiovascular health centers in eight states in the Northeast region. Food consumption was obtained by 24-hour dietary records and dietary adequacy followed SBC recommendations. Values of p < 0.05 were considered significant.

**Results:**

647 patients were studied, with a mean (standard deviation) age of 63.1 (9.4) years, 50.2% of whom were female. When evaluating food intake, a low adequacy of carbohydrates (52.3%), proteins (70.9%), lipids (38.8%), and fiber (22.4%) was observed. It was observed that the majority of women consumed a low-protein diet (59.2%) and the elderly had a greater inadequacy in carbohydrate consumption (52.6%). Regarding sodium intake, men had a higher intake (72.9%), while the elderly showed a 13% reduction. Furthermore, it was shown that men ate more fiber (28.1%) and individuals with higher education had a high consumption of saturated fatty acids (70.5%).

**Conclusions:**

Most individuals did not achieve the recommended dietary therapy goals for secondary cardiovascular prevention. The findings of the present study reinforce the need to implement structured strategies to encourage healthy eating habits in these individuals.

## Introduction

Data from the World Health Organization demonstrate that cardiovascular diseases (CVD) are the leading cause of mortality in the world.^1^ In Brazil, a similar scenario is observed, in which these diseases were responsible for approximately 27% of all deaths in 2019.^2^ This data is alarming, as approximately 20% of individuals who had a cardiovascular event suffer a second one a year later,^3^ and secondary prevention is considered a key point to effectively achieve the therapeutic goals of cardiovascular protection.^4-7^

Secondary cardiovascular prevention is defined as a set of preventive strategies, aiming to stop the progression of the disease.^8^ Adopting healthy eating habits and lifestyle changes are among the important strategies for preventing recurrent cardiovascular events.^8,9^ Considering this context, the Brazilian Cardioprotective Nutritional (BALANCE) was developed, called Brazilian Cardioprotective Diet (DICA BR),^10,11^ a food program consisting of dietary prescription with adaptations of the Mediterranean diet through the valorization of foods feasible for the Brazilian population, based on nutritional recommendations. of the guidelines of the *Sociedade Brasileira de Cardiologia.*^12^

Although studies demonstrate the importance of nutrition in secondary cardiovascular prevention, achieving the therapeutic goals established by scientific societies is a constant challenge and not always achieved.^5^ Despite extensive knowledge about the importance of reducing cardiovascular risk factors, a high proportion of individuals with CVD do not adequately control these factors.^5,10^

Although a reduction in mortality from CVD^1^ has been observed worldwide, death rates from these diseases remain high in some Brazilian regions, particularly the Northeast region of the country.^2^ However, studies that evaluate secondary prevention measures in this region are scarce, so the objective of the present study was to investigate the dietary adequacy of individuals with cardiovascular disease (CVD), participants in the BALANCE Program, residents of the Northeast region of Brazil, according to recommendations of the *Sociedade Brasileira de Cardiologia*.

## Methods

### Study design

This is a cross-sectional study, part of a broader work, conducted with data from patients of the BALANCE Program^11^ implementation study. The data collection process was carried out at baseline between 2013 and 2015, by previously trained nutritionists. A detailed description of the study design has been previously published.^12^

### Sample

The sample was made up of patients treated in ten cardiovascular health reference centers located in the Northeast Region of Brazil. The inclusion criteria adopted in the DICA BR study were: age equal to or greater than 45 years, both sexes and having evidence of atherosclerosis (coronary artery disease, cerebrovascular disease, or peripheral arterial disease), currently or in the last ten years diagnosed by the attending physician.

Patients were considered to have coronary artery disease (CAD) if they presented one or more of the following characteristics: asymptomatic CAD (history of coronary angiography or coronary tomography angiography with atherosclerotic stenosis ≥70% of the diameter of any coronary artery); symptomatic CAD (history of angina: clinical diagnosis, even without additional tests; history of positive stress test); Treated CAD (previous angioplasty/Stent/revascularization) and Infarction (history of myocardial infarction or acute coronary syndrome; history of abnormality in the segmental movement of the heart wall on echocardiography or a fixed segmental defect on scintigraphy).

In turn, the diagnosis of cerebrovascular disease (Ischemic Stroke-IS / Transient Ischemic Attack-TIA / Cerebral Vascular Accident-CVA) was considered when the patient presented one or more of the following criteria: medical diagnosis of stroke or TIA; evidence of previous stroke on computed tomography or magnetic resonance imaging.

Regarding the diagnosis of peripheral arterial disease (PAD), it was considered when the individual presented one or more of the following criteria: asymptomatic PAD (ankle/arm ratio <0.9 systolic blood pressure in either leg at rest; angiographic study or Doppler demonstrating >70% stenosis in a non-cardiac artery); symptomatic PAD (intermittent claudication); treated PAD (vascular surgery for atherosclerotic disease); Amputation due to arterial causes and aortic aneurysm.

The non-inclusion criteria were: psychiatric and/or neurocognitive condition that prevented data collection; life expectancy of less than six months; pregnancy or lactation; liver failure with a history of encephalopathy or anasarca; renal failure requiring dialysis; Congestive heart failure; organ transplantation; gastroplasty; wheelchair users and participants who had difficulty feeding orally.

### Data collection

The data obtained for this work were: smoking, physical inactivity, hypertension (SAH), diabetes mellitus (DM), dyslipidemia, treated coronary artery disease (treated CAD and PAD), and blood pressure measurement, as described in the literature.^13^ Regarding socioeconomic variables, education was classified as lower (illiterate or elementary school) or higher level (high school or higher). As for social class, it was classified as lower (C, D, and E) or higher (A and B).^14^

### Laboratory tests

The analysis of laboratory tests was carried out with the patient fasting for 12 hours and no alcohol consumption in the previous 72 hours. Traditional cardiovascular risk markers were used: triglycerides, total cholesterol, high-density lipoproteins (HDL-c), low-density lipoproteins (LDL-c), and non-HDL cholesterol, classified according to recommended recommendations.^7,15^

### Anthropometric assessment

Weight and height data were used to calculate the variable body mass index (BMI). For analysis purposes, individuals were grouped as being underweight, when their BMI was less than 18.5kg/m^2^ and 23.0kg/m^2^; eutrophic with values between 18.5-24.9kg/m^2^ and 23.0-27.9kg/m^2^; excess weight when the BMI was equal to or greater than 25.0kg/m^2^ and 28.0kg/m^2^, respectively in adults and the elderly. Obesity was defined as a BMI equal to or greater than 30.0kg/m^2^, for both.^16,17^ The presence of central obesity was determined by measuring waist circumference, according to the recommended technique and cutoff points.^18^

### Assessment of food intake

For the analysis of food intake, the 24-hour food record (R24H) was used, which was obtained according to the multiple-pass methodology,^19^ with the use of a photographic album of food measurements and portions, aiming to increase the reliability of the information collected. To analyze the nutritional composition of the R24H obtained, the Nutriquanti® program was used, which prioritizes Brazilian nutritional composition tables.

The amount of total energy value (TEV) and proteins ingested was expressed per kilogram of weight. For overweight and obese individuals, energy consumption in the range of 20.0 to 25.0kcal/kgP/day was considered adequate.^20^ However, given the lack of recommendation from the *Sociedade Brasileira de Cardiologia* for energy intake in individuals with atherosclerosis with low weight and normal weight, 30.0 to 35.0kcal/KgP and 25.0 to 30.0kcal/KgP were established as adequate. Respectively.^21^

The intake of macronutrients (carbohydrates and lipids) was evaluated as a percentage using the TEV consumed as a reference. Consumption of protein (equal to or greater than 0.8g/KgP),^20^ sodium (less than 2,000mg),^13^ carbohydrates (45 to 60%), dietary fiber (equal to or greater than 25g), lipids (25 to 35%), polyunsaturated fatty acids (PUFA) from 5 to 10%, monounsaturated (MUFA) 15%, saturated (SFA) less than 7% and absence of trans fatty acids.^7^

Nutritional adjustments regarding the consumption of calcium, magnesium, and potassium, due to the lack of specific recommendations for individuals with CVD, were considered according to sex and age group based on the EAR (Estimated Average Requirements) micronutrient intake recommendations, through the Dietary reference intakes (DRI).^22^

### Ethics Committee

The study was conducted under ethical principles and the study protocol was approved by the Human Research Ethics Committee of Hospital do Coração (Hcor), as well as the ethics committees of each collaborating center. All participants signed the Free and Informed Consent Form (TCLE).

### Statistical analysis

For data analysis, the Statistical Package for the Social Sciences (SPSS) program, version 17, was used. Data were presented by simple and relative frequency, mean and standard deviation (SD) or median (Md), and interquartile range (IIQ), according to the nature of the variables. The normality of quantitative variables was tested using descriptive statistics, graphical analysis, and normality tests (Kolmogorov-Smirnov). The associations were tested using the prevalence ratio with respective 95% confidence intervals (95% CI), in which the value 1 was assigned to the reference category in the prevalence ratio analysis. The individuals were divided into two groups, “within the yes or no recommendation” for comparison purposes, using Pearson’s chi-square test to compare proportions. Values of p < 0.05 were considered significant.

## Results

654 individuals were selected, seven of whom were not included in the sample because they did not attend the initial consultation, totaling 647 patients. Of the nine states that make up the Northeast region of Brazil, only the state of Piauí did not participate in the study. The patients had a mean (SD) age of 63.1 (9.3) years, with a predominance of females, elderly people, individuals with a low level of education, and sedentary (Table 1).

**Table 1:** – Sociodemographic, lifestyle, clinical, and anthropometric characteristics of 647 individuals with cardiovascular disease, followed up at reference cardiovascular care outpatient clinics in eight states in the Northeast of Brazil

Variables	Results
**Sociodemographic and lifestyle**	
Age (years)*	63,1 (9,3)
Female	325 (50,2%)
**Education level**	
Illiterate	210 (37,6%)
Fundamental	211 (37,8%)
High school	100 (17,9%)
Higher	37 (6,6%)
**Social class**	
Class A and B	112 (20,0%)
Class C, D, and E	447 (80,0%)
Sedentary lifestyle	463 (73,3%)
Ex-smoking	342 (53,0%)
Smoking	27 (4,2%)
**Clinics**	
Dyslipidemia	625 (96,6%)
Coronary artery disease	599 (92,6%)
Arterial hypertension	580 (89,6%)
Acute myocardial infarction	294 (45,4%)
Diabetes mellitus	261 (40,3%)
Stroke	111 (17,2%)
Peripheral arterial disease	61 (9,4%)
Treated coronary artery disease	425 (65,7%)
**Anthropometry**	
Underweight	47 (7,3%)
Eutrophic	229 (35,7)
Overweight	177 (27,6%)
Obesity	188 (29,3%)
Abdominal obesity	486 (77,0%)
**Achievement of therapeutic goals**	
LDL-c	45 (7,8%)
Non-HDL-c	57 (9,8%)
Triglycerides	341 (58,6%)

*Data in mean and standard deviation.

In the assessment of food intake, 49 individuals did not have a food record at baseline, therefore, the analysis of nutritional composition was carried out in 598 individuals. Inadequacies in the consumption of macronutrients, fiber, and micronutrients were observed, as described in Table 2.

**Table 2:** – Adherence to nutritional goals for secondary cardiovascular prevention in 598 individuals with cardiovascular disease treated in ten outpatient clinics specialized in cardiovascular health in eight states in the Northeast Region of Brazil

Variables	Results	Within Recommendations
Calorie rate (Kcal/KgP)	24.7 (22.6 - 28.0)	111 (18.6%)
Carbohydrates (%)	55.6 (10.6)	313 (52.3%)
Protein (g/KgP)	1.0 (0.7 - 1.3)	424 (70.9%)
Lipids (%)	24.6 (7.6)	232 (38.8%)
Trans fatty acids (%)	0.02 (0.01 - 0.03)	598 (100.0%)
Saturated fatty acids (%)	7.8 (5.7 - 10.6)	244 (40.8%)
MUFA (%)	6.9 (5.3 - 8.7)	14 (2.3%)
PUFA (%)	0.6 (0.5 - 0.8)	-
Fibers (g)	16.5 (11.4 - 23.2)	134 (22.4%)
Sodium (mg)	2,384.3 (1,720.6 - 3,180.6)	219 (36.6%)
Potassium (mg)	2,037.0 (1,491.1 - 2,649.3)	2 (0.3%)
Magnesium (mg)	182.2 (132.1 - 239.5)	24 (4.0%)
Calcium (mg)	471.0 (271.7 - 757.1)	45 (7.5%)

*MUFA: monounsaturated fatty acids; PUFA: polyunsaturated fatty acids.*

Tables 3 and 4 describe the prevalence of inadequacies in food consumption and prevalence reasons, according to gender, age group, and sociodemographic variables. The summary of the study findings can be seen in the Central Illustration.

**Table 4:** – Prevalence and gross prevalence ratios of inadequacies in the consumption of fiber, sodium, and saturated fatty acids, according to gender, age group, and sociodemographic variables, of individuals with atherosclerosis followed in ten reference cardiovascular health clinics in eight cities in the Northeast Region

Variables	Low fiber consumption				Sodium high consumption				SFA high consumption			
	n	p (%)	PR	CI 95%	n	p (%)	PR	C I95%	n	p (%)	PR	CI (95%)
**Sex**												
Male	210	71,9	0,87	0,79 - 0,95	213	72,9	1,34	1,19 - 1,52	176	60,3	1,04	0,91 - 1,18
Female	254	83,0	1,0		166	54,2	1,0		178	58,2	1,00	
**Elderly**												
Yes	284	78,7	1,04	0,95 - 1,13	216	59,8	0,87	0,77 - 0,98	207	57,3	0,92	0,81 - 1,06
No	180	75,9	1,0		163	68,8	1,0		147	62,0	1,00	
**Education**												
Lower	313	78,6	1,09	0,97 - 1,23	245	61,6	0,87	0,76 - 1,00	225	56,5	0,82	0,71 - 0,95
Higher	95	72,0	1,0		93	70,5	1,0		91	68,9	1,00	
**Social class**												
Lower	331	78,4	1,09	0,96 - 1,23	266	63,0	0,94	0,81 - 1,10	244	57,8	0,86	0,73 - 1,00
Higher	78	72,2	1,0		72	66,7	1,0		73	67,6	1,00	

*SFA: Saturated Fatty Acids; PR: prevalence ratio; CI: confidence interval.*

**Table 3:** – Prevalence and crude prevalence ratios of inadequacies in the consumption of calories and macronutrients, according to gender, age group, and sociodemographic variables, of individuals with atherosclerosis followed up in ten reference cardiovascular health outpatient clinics in eight cities in the Northeast Region

Variables	Inadequate calorie rate				Inadequate carbohydrate intake				Low protein intake			
	n	p (%)	PR	CI 95%	n	p (%)	PR	CI 95%	n	p (%)	PR	CI (95%)
**Sex**												
Male	230	78.8	0.94	0.87-1.01	140	47.9	1.01	0.86-1.20	71	40.8	0.72	0.56-0.93
Female	256	83.9	1.00		145	47.4	1.00		103	59.2	1.00	
**Elderly**												
Yes	292	81.1	0.99	0.92-.,07	190	52.6	1.31	1.09-1.58	100	57.5	0.89	0.69-1.14
No	194	81.9	1.00		95	40.1	1.00		74	42.5	1.00	
**Education**												
Lower	329	82.9	1.07	0.97-1.19	191	48.0	0.93	0.75-1.16	123	77.4	0.88	0.64-1.21
Higher	102	77.3	1.00		59	44.7	1.00		36	22.6	1.00	
**Social class**												
Lower	350	82.9	1.08	0.97-1.21	203	48.1	0.91	0.71-1.15	129	81.1	0.91	0.65-1.27
Higher	82	76.6	1.00		47	43.5	1.00		30	18.9	1.00	

*PR: prevalence ratio; CI: confidence interval.*

## Discussion

In the present study, it was found that most individuals were highly inadequate in meeting the dietary goals established for secondary cardiovascular prevention. In other studies, relevant inadequacies in nutritional goals were also found, corroborating the results presented.^23,24^ Furthermore, it was found that elderly individuals had a greater inadequacy of carbohydrate consumption, those with higher education had a high consumption of SFA, in addition, men had a higher intake of sodium, and women had a greater inadequacy of fiber. These data draw attention, as individuals with these characteristics are likely to show low dietary adequacy. It is worth mentioning that dietary inadequacies may have influenced the failure to meet therapeutic goals related to lipid profile^7^, blood pressure,^13^ and glycemic control.^15^

The *Sociedade Brasileira de Cardiologia* and the American Heart Association recommend that the joint application of strategies related to drug and non-pharmacological therapy in the management of atherosclerosis is potentially beneficial.^4,6,7^ Previous studies have investigated the association of dietary patterns with recurrent cardiovascular events^11,25^ and that the adoption of healthy eating habits seems to act synergistically with the control of cardiovascular risk factors.^26^

“Atherogenic” food consumption, evidenced by the high consumption of SFA and sodium, predominated in the individuals studied. Evidence demonstrates possible nutrients identified as risk factors for CVD, including high consumption of sodium, SFA, trans fat,^27^ and refined carbohydrates.^28^ In contrast, reducing the consumption of foods that are sources of these nutrients has proven to be extremely important in the prevention and control of CVD.^29^

When analyzing the food intake of the studied sample, it was observed that few individuals were consuming the number of calories necessary for their nutritional status. The literature demonstrates possible factors that make it difficult to achieve nutritional recommendations, including socioeconomic issues,^30^ and lack of knowledge or poor adherence to nutritional guidelines.^31^

It is important to highlight that evaluating the quality of the diet is extremely relevant. In the present study, despite most individuals having a low-fat diet, a high consumption of SFA and a low intake of unsaturated fatty acids was observed, corroborating other authors.^32^ Studies have demonstrated that qualitative changes in the lipid composition of the diet seem to have beneficial effects in reducing cardiovascular events and mortality, although the results are conflicting.^27^ In a meta-analysis, conflicting results were observed from lipid-reduced diets and the replacement of SFA by PUFA.^33^ This can be attributed to the fact that the exchange of SFA for unsaturated fatty acids, in addition to reducing LDL-c, can reduce HDL-c, occurring more intensely when replacing SFA with MUFA.^34^

Among strata with higher education, the level of education is associated with greater SFA intake. Therefore, when analyzing education as an indirect measure of income, it is observed that individuals with a more favorable socioeconomic level have a higher prevalence of inadequate consumption of SFA, corroborating other authors.^35^ In this context, it is also worth highlighting that, in the present study, approximately 30% of the individuals had a low-protein diet. It is known that these foods usually have a higher cost and are less available among lower-income individuals.^36^

It was evidenced that a small proportion of individuals met the dietary fiber recommendation, a similar finding was found by other studies,^23,37^ possibly due to the adoption of unhealthy eating habits, low quality of carbohydrate intake,^28^ and high consumption of ultra-processed foods identified by high sodium consumption.^37^ Regarding differences in dietary fiber consumption between the sexes, women had a higher prevalence of inadequacy. Studies have shown that dietary fiber consumption reduces proportionally in the presence of worse socioeconomic conditions, which could justify the low consumption seen among women^36^. A meta-analysis demonstrated that fiber intake was associated with a beneficial reduction in total cholesterol, LDL-c, and diastolic blood pressure.^38^

High sodium consumption was higher among men, a finding similar to that of other authors.^39^ According to the Family Budget Survey (FBS), daily sodium consumption in Brazil is approximately 4,700 mg.^40^ Because sodium intake is considered an important marker of diet quality in secondary cardiovascular prevention, the need to apply prevention actions is reinforced, aiming to reduce sodium consumption.^24^

Still concerning food consumption, potassium, magnesium, and calcium are essential nutrients, which play an important role in preserving vascular tone and cardiac contractility.^23^ The consumption of these micronutrients in the present study was extremely low, which reflects the low quality of the diet, which can be justified by the inadequate consumption of foods that are sources of vitamins and minerals, in addition to the predominance of individuals with low purchasing power, favoring less access to a more varied and healthier diet.^36^

Among the limitations of this study, we can mention the lack of more accurate methods to assess nutritional status. However, BMI and waist circumference were assessed together to minimize this limitation. It is noteworthy that these indicators are low-cost and easy to apply in clinical practice. Furthermore, it must be considered that memory bias may have occurred, given that the R24H is an instrument dependent on the interviewee’s memory. The limitations of using this instrument lie in the fact that food intake on a single day may not reflect the individual’s usual diet. However, we believe that these individuals have low variability in food consumption since the sample is predominantly composed of individuals from lower social classes.

It is important to highlight that given the absence of specific recommendations for individuals with CVD, the adequacy of micronutrient intake was carried out based on the DRI. However, these recommendations were developed for a healthy, non-Brazilian population, so the findings should be analyzed with caution.

BALANCE Program is the first national study working on regionality and nutrition, relating them to cardiovascular health. It is noteworthy that the program is unprecedented in the country, as it considers the food culture of Brazilian regions. Adhering to healthy eating habits is a challenging and dynamic process for both patients and healthcare professionals.^31^ Given the success of the First Brazilian Cardioprotective Diet, a guidance manual for Primary Care health professionals^41^ was prepared by the Ministry of Health, with nutritional guidelines for individuals with cardiovascular risk factors.

Adequate dietary intervention allows for a better combination of multiple foods and nutrients, with cardioprotective properties in the secondary prevention of CVD. Therefore, it is important to encourage greater consumption of these nutrients, favoring better control of cardiovascular risk factors. It is worth highlighting the fact that this work is a regional study, aiming to provide relevant information to health professionals about the profile of northeastern individuals with CVD, as well as identifying factors that may influence nutritional adequacy in secondary cardiovascular prevention.

## Conclusions

The results of the study indicated that most individuals did not achieve the dietary therapy goals recommended for secondary cardiovascular prevention, according to the *Sociedade Brasileira de Cardiologia*. Therefore, they had a diet low in fiber, micronutrients, and low nutritional quality, with a high intake of saturated fat and low intake of unsaturated fatty acids, all of which predisposed the occurrence of other cardiovascular outcomes. Excessive sodium consumption was identified, especially in males, which is a worrying factor, as these individuals, due to their very high cardiovascular risk, require lower amounts of this nutrient.
